# A retrospective study of treatment persistence and adherence to mirabegron versus antimuscarinics, for the treatment of overactive bladder in Spain

**DOI:** 10.1186/s12894-018-0390-z

**Published:** 2018-09-04

**Authors:** Jameel Nazir, Zalmai Hakimi, Florent Guelfucci, Amine Khemiri, Francis Fatoye, Ana María Mora Blázquez, Marta Hernández González

**Affiliations:** 1 Astellas Pharma Europe Ltd, 2000 Hillswood Drive, Chertsey, KT16 0PS UK; 2 Astellas Pharma Europe B.V, Leiden, the Netherlands; 3 Creativ-Ceutical Ltd., London, UK; 4 Creativ-Ceutical Ltd., Tunis, Tunisia; 5 Manchester Metropolitan University, Manchester, UK; 6 Astellas Pharma S.A., Madrid, Spain; 7 Present Address: Keyrus Biopharma, Tunis, Tunisia

**Keywords:** Overactive bladder, Mirabegron, Antimuscarinics, Treatment persistence

## Abstract

**Background:**

Persistence on-treatment with antimuscarinics in patients with overactive bladder (OAB) is reported to be sub-optimal. This retrospective, longitudinal, observational cohort study assessed treatment persistence with β_3_-adrenoceptor agonists (i.e. mirabegron) and antimuscarinics, both classes of OAB pharmacotherapy, in patients with OAB in Spain.

**Methods:**

Adults who received mirabegron or an antimuscarinic in routine clinical practice (1 June*–*31 October 2014), were identified from anonymised prescription data within the Spanish Cegedim Electronic Medical Records database. The primary endpoint, treatment persistence (time to treatment discontinuation [TTD] and the proportion of patients remaining on-treatment after 12 months), was unadjusted for potential confounders. Multivariate Cox regression models of persistence, adjusted for baseline characteristics, were used to compare differences in treatment groups. Adjusted subgroup analyses (target OAB drug, age, treatment status and sex) and sensitivity analyses (extending the time used to define treatment discontinuation from 30 days [base-case] to 45, 60 or 90 days without prescription renewal) were also performed.

**Results:**

Overall, 1798 patients received mirabegron (*N* = 1169) or an antimuscarinic (*N* = 629); the mean age was 66.42 years. Median TTD was longer for mirabegron versus antimuscarinics (90 vs 56 days) and a higher proportion of patients who received mirabegron were persistent after 12 months (20.2% vs 10.2%); multivariate analyses indicated significantly greater persistence with mirabegron versus antimuscarinics (hazard ratio [HR]: 1.52; 95% confidence interval [CI]: 1.37–1.70; *p* < 0.001). Significant differences were also observed in subgroup analyses of mirabegron versus individual antimuscarinics (median TTD: 90 vs [range] 28–60 days; HR range: 1.21–2.17; *p* ≤ 0.013) and in all other subgroups assessed (*p* < 0.001). Sensitivity analysis showed that the median TTD for mirabegron increased by up to 31 days, and was significantly longer versus antimuscarinics across all adjusted periods (HR range: 1.43–1.53; all *p* < 0.001).

**Conclusions:**

Patients with OAB in Spain who received mirabegron experienced longer persistence on-treatment than those who received antimuscarinics and the proportion of patients persistent on-treatment at 12 months with mirabegron was two-times higher versus antimuscarinics. These data may provide strategic insights for clinicians and policy makers involved in the management of OAB.

**Electronic supplementary material:**

The online version of this article (10.1186/s12894-018-0390-z) contains supplementary material, which is available to authorized users.

## Background

Overactive bladder (OAB) is characterised by urinary urgency, usually accompanied by frequency and nocturia, with or without urgency urinary incontinence, in the absence of urinary tract infection or other obvious pathology [1, 2]. The prevalence of OAB in Spain is approximately 20% in patients ≥40 years of age; OAB is most common in the elderly population, observed in more than one-quarter of Spanish adults >70 years of age [3].

Health-related quality of life (HRQoL) is profoundly affected in patients who experience OAB symptoms, as demonstrated by clinically meaningful impairments in HRQoL scores [4]. Aspects of life affected by OAB symptoms include sexual health, personal relationships, performing daily activities [5, 6], employment and productivity in the workplace [6, 7]. The major impact of symptoms is exemplified by a study of OAB in Spain, which reported that 96% of patients adopted non-medical coping strategies to help manage their daily and occupational activities, and up to 10% of work time was lost by patients experiencing urgency and urinary incontinence (UI) [8]. OAB is also associated with a substantial economic burden; the total annual direct economic impact (incremental costs) of OAB on the Spanish National Healthcare System is estimated to be around €366 million [7].

Following the use of conservative management strategies (recommended as initial treatment for OAB), oral pharmacotherapy with β_3−_adrenoceptor agonists or antimuscarinic agents can improve OAB symptoms [9]. However, evidence from a large, retrospective analysis of medical and pharmaceutical claims database (*N* = 167,907) suggests that persistence (i.e. the duration of time from initiation to discontinuation of therapy [10]) and adherence (i.e. the extent to which a patient acts in accordance with the prescribed interval, and dose of a dosing regimen [10]) to antimuscarinics are low compared with pharmacological therapies used to treat other chronic diseases, including statins and oral diabetics [11]. A systematic literature review (SLR) and meta-analysis that assessed persistence and adherence to antimuscarinic therapy for OAB (14 retrospective database or self-report studies; *N* = 190,279) reported 12-month median persistence rates of 12.0*–*39.4%; persistence was evaluated up to 36 months and decreased over time [12]. Data from a review of 14 retrospective medical claims studies suggest that a high proportion (43–83%) of patients with OAB discontinued antimuscarinic treatment within the first 30 days [13]. In addition to poor tolerability, unmet treatment expectations, patient education and costs are cited as common reasons for discontinuing antimuscarinic treatment [14].

Mirabegron is a first-in-class licensed, selective, oral β_3−_adrenoceptor agonist, which was approved for the treatment of OAB in Europe in December 2012 [15] and was first marketed in Spain in April 2014 [16]. Improved efficacy and similar tolerability for mirabegron compared with placebo has been demonstrated in several phase III studies [17–19]. Similar overall efficacy and improved tolerability regarding bothersome anticholinergic adverse events (AEs) was reported for mirabegron versus antimuscarinics in a network meta-analysis of 44 randomised controlled trials of OAB (*N* = 27,309) [20].

Significant improvements in persistence and adherence have been reported for mirabegron compared with antimuscarinics, in two large retrospective observational studies conducted in Canada and the United Kingdom (UK) [21, 22]. Persistence was also significantly greater for mirabegron versus tolterodine in a large, retrospective analysis of claims records of patients in the United States [23]. These findings may be due, in part, to a lower incidence of bothersome anticholinergic AEs, such as dry mouth, reported for mirabegron compared with antimuscarinics [20], which are frequent reasons for treatment discontinuation [24–26]. Other factors such as the information provided for the patient about their condition (i.e. patient education), patients’ willingness to take long-term treatment, and treatment costs may also affect persistence and adherence in patients with OAB [14, 27–29].

Persistence and adherence in patients with OAB are associated with several clinical and economic benefits. Significant improvements in OAB symptoms and HRQoL have been reported in patients adherent to and those persistent on-treatment, respectively, compared with non-adherent or non-persistent patients [30, 31]. Moreover, economic models of OAB suggest that improvements in persistence with mirabegron versus antimuscarinics translate into reduced healthcare resource use, fewer lost work hours, and lower total costs [32, 33].

Data are available from two retrospective, observational studies which evaluated treatment persistence and adherence in patients receiving pharmacotherapy for OAB in Spain [34, 35]. However, limitations in the design of these studies include possible under-reporting of the incidence of OAB and a lack of evaluation of other variables besides pharmacological treatment. Multivariate analyses to adjust for the impact of baseline characteristics were included in the current study, which assessed treatment persistence and adherence observed with mirabegron versus several common antimuscarinics in patients with OAB in Spain.

## Methods

### Study design and objectives

This was a retrospective, longitudinal, observational cohort study of patients with OAB who received mirabegron or an antimuscarinic drug in routine clinical practice. Anonymised prescription records were collected from the Spanish Cegedim Electronic Medical Records database, which holds information for approximately one million patients, submitted by primary and secondary care physicians in Spain.

The primary objective was to compare treatment persistence with mirabegron versus antimuscarinics in all eligible patients with OAB. Secondary objectives included: comparing treatment adherence with mirabegron versus antimuscarinics in all eligible patients; evaluating treatment persistence and adherence in different subgroups; and investigating the impact of different patient characteristics on treatment persistence and adherence.

Ethics committee approval for the study was obtained from the Hospital Clinic of Barcelona.

### Study population

Eligible patients were ≥ 18 years of age and received a prescription for a target OAB drug between 1 June and 31 October 2014 (i.e. the selection period); the date of the first prescription of that drug was the index date (Additional file 1: Figure S1). Eligible patients were also required to have continuous enrolment during the pre- and post-index periods (12 months prior to and 12 months after the index date, respectively). Exclusion criteria were: prior prescription of the target OAB drug or a 5α-reductase inhibitor during the pre-index period, and prescription of another OAB drug (i.e. receipt of concomitant therapy) at the index date or up to 30 days afterwards; concomitant therapy was permitted > 30 days after the index date. Eligible drugs were identified using European Pharmaceutical Market Research Association (EphMRA) Anatomical Therapeutic Chemical (ATC) codes [36] (Additional file 2: Table S1).

### Endpoints

Treatment persistence, the primary endpoint, was defined as the time from the index date until first discontinuation of the index drug (i.e. time to discontinuation [TTD]). An index drug was considered discontinued after a period of 30 days without prescription renewal.

Adherence, determined using the medical possession ratio (MPR), was assessed as a secondary endpoint. This was calculated by two methods: the sum of days’ supply of the index drug, divided by 365 days (i.e. fixed-MPR), or divided by the TTD (i.e. variable-MPR). Patients were considered adherent with a MPR of ≥80%.

### Statistical analyses

Treatment persistence and adherence (primary and secondary endpoints), assessed in all eligible patients, were not adjusted for potential confounding factors and data were reported descriptively. The unadjusted analyses of persistence were presented using Kaplan-Meier curves and reported as median TTD and the proportion of patients persistent at the end of the 12-month post-index period. The unadjusted analyses of adherence were reported as mean MPR and the proportion of adherent patients at the end of the 12-month post-index period.

Multivariate analyses using Cox and linear regression models were used to analyse TTD and MPR, respectively. The target OAB drug (i.e. mirabegron or antimuscarinics) was the independent variable; adjustments were made for potential confounding baseline factors of treatment status (naïve or experienced) and age (45–64, 65–74 or ≥ 75 years for analyses of TTD; < 65 or ≥ 65 years for evaluation of MPR). Treatment-naïve patients received no prescriptions for an OAB drug during the 12 months prior to the index date, while treatment-experienced patients received prior prescription(s) for a different OAB drug to the index drug. The models were fitted by including all covariates that showed a significant relationship (*p* < 0.10) with TTD or fixed- and variable-MPR in univariate Cox or linear regression models. Additionally, a forward selection process was used in the multivariate analyses and any variables which did not maximise the quality of the models were excluded [37, 38].

Analyses of treatment persistence and adherence for mirabegron versus antimuscarinics (also using Cox and linear regression models, respectively) were performed, considering each individual antimuscarinic, and in the following patient subgroups: treatment-naïve or -experienced; < 65 or ≥ 65 years of age; and male or female. In addition, sensitivity analyses were performed to assess the impact of increasing the period without prescription renewal used to define TTD from 30 days (base case) to 45, 60 and 90 days. The sensitivity analyses were also adjusted for baseline characteristics.

Adjusted analyses were reported as hazard ratios (HRs) with 95% confidence intervals (CI) and *p*-values for TTD, or *p*-values only for MPR. For all adjusted comparisons, mirabegron was the reference comparator and a *p*-value of < 0.05 was considered statistically significant.

All analyses were performed using Statistical Analysis Software, version 9.3.

## Results

### Baseline demographics and characteristics

**Fig. 1:**
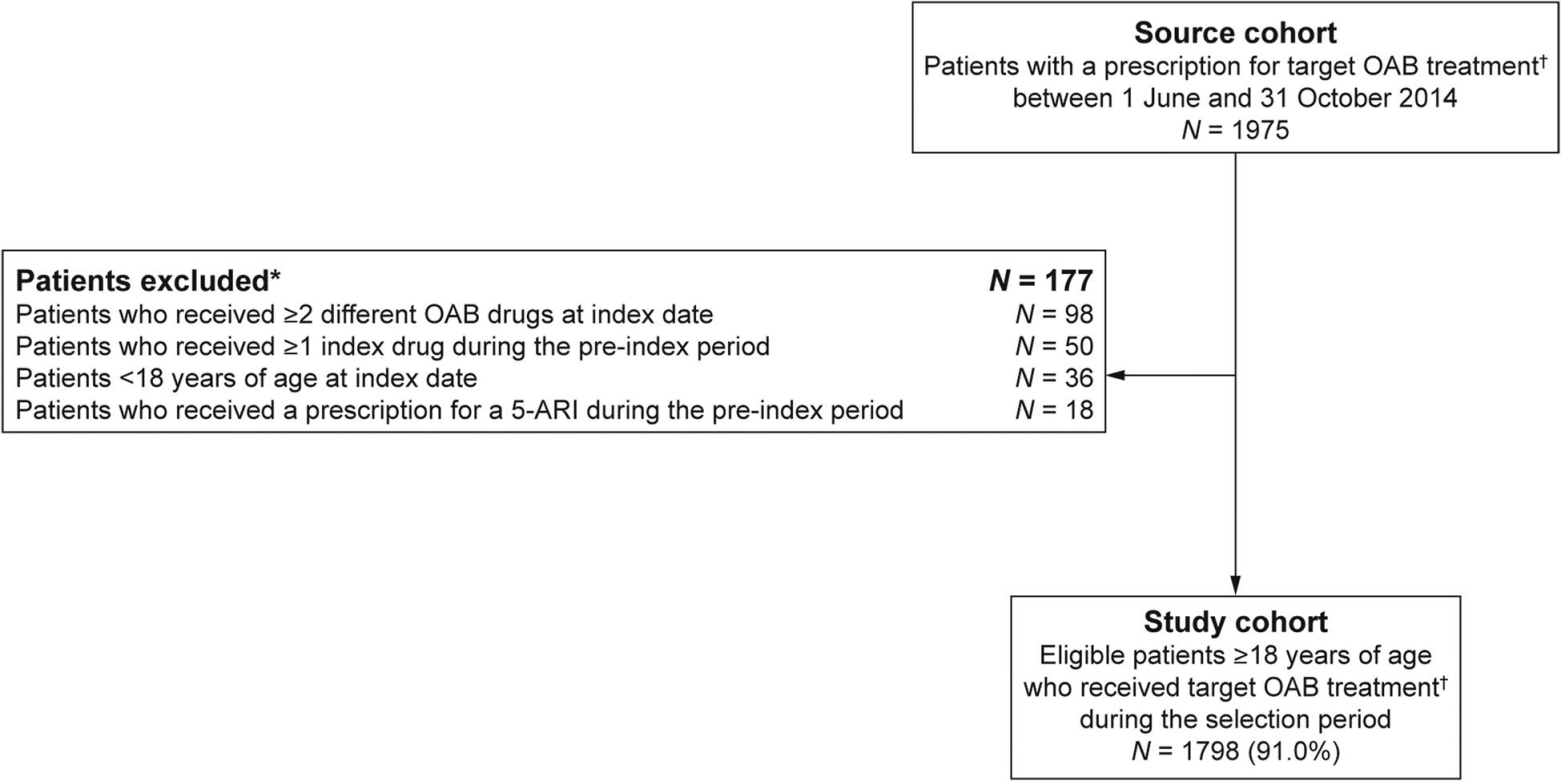
Patient selection flowchart.^*^Multiple reasons for exclusion may have applied for a single patient, therefore 202 reasons for exclusion are listed; no patients were excluded because of an insufficient pre-index period (i.e. < 12 months’ history) or an insufficient post-index period (i.e. < 12 months’ follow-up); ^†^Mirabegron or an antimuscarinic; eligible drugs listed in Additional file 6: Table S1. 5-ARI, 5α-reductase inhibitor; OAB, overactive bladder

Overall, 1975 patients received a prescription for a target OAB drug during the selection period and 1798 were eligible for inclusion (Fig. 1). The most common reasons for exclusion were receipt of two or more different OAB drugs (i.e. concomitant therapy) at index date or up to 30 days afterwards (*N* = 98), receipt of the index drug during the pre-index period (*N* = 50), and < 18 years of age at the index date (*N* = 36). A total of 1169 (65.0%) eligible patients received mirabegron and 629 (35.0%) received an antimuscarinic. Solifenacin was the most common prescribed antimuscarinic (*N* = 266; 14.8% of patients).

The mean age at index date was 66.42 years (Table 1); a higher proportion of patients were 65–80 years old in the mirabegron arm compared with the antimuscarinic arm (52.8% vs 44.0%), but a lower proportion were > 80 years old (11.8% vs 18.6%). Overall, 1021 (56.8%) patients were female; 637 (54.3%) and 384 (61.0%) in the mirabegron and antimuscarinic arms, respectively. Body mass index (BMI) data were available for 1185 patients, of whom, 545 (46.0%) were obese and 430 (36.3%) were overweight. Of 1289 (71.7%) treatment-naïve patients, a higher proportion was observed in the antimuscarinic arm compared with the mirabegron arm (85.7% vs 64.2%). Approximately two-thirds of patients in the mirabegron arm received their prescription in secondary care from a gynaecologist/urologist; around three-quarters of patients treated with an antimuscarinic received their prescription in primary care, from a general practitioner (GP).

**Table 1:** Baseline characteristics in patients who received prescriptions for mirabegron or antimuscarinics^a^

	Mirabegron (*N* = 1169)	Antimuscarinics (*N* = 629)	Total (*N* = 1798)
Sex, *N* (%)			
Male	532 (45.5)	245 (39.0)	777 (43.2)
Female	637 (54.5)	384 (61.0)	1021 (56.8)
Age, mean (SD)	66.31 (13.41)	66.61 (14.71)	66.42 (13.88)
Age category, *N* (%)			
< 65 years	414 (35.4)	235 (37.4)	649 (36.1)
65–80 years	617 (52.8)	277 (44.0)	894 (49.7)
> 80 years	138 (11.8)	117 (18.6)	255 (14.2)
BMI, mean (SD)	30.15 (5.47)	29.88 (5.62)	30.06 (5.52)
BMI category, *N* (%)^b^			
Underweight (< 18.5)	4 (0.5)	5 (1.3)	9 (0.8)
Normal or healthy weight (18.5–24.9)	137 (17.3)	64 (16.2)	201 (17.0)
Overweight (25.0–29.9)	277 (35.1)	153 (38.7)	430 (36.3)
Obese (≥30.0)	372 (47.1)	173 (43.8)	545 (46.0)
Treatment status, *N* (%)			
Treatment-naïve	750 (64.2)	539 (85.7)	1289 (71.7)
Treatment-experienced	419 (35.8)	90 (14.3)	509 (28.3)
Prior treatment with α-blockers, *N* (%)^c^			
Yes	157 (13.4)	58 (9.2)	215 (12.0)
No	1012 (86.6)	571 (90.8)	1583 (88.0)
Prescriber at index date, *N* (%)			
Gynaecologist/urologist	749 (64.1)	131 (20.8)	880 (48.9)
GP	397 (34.0)	476 (75.7)	873 (48.6)
Other	23 (2.0)	22 (3.5)	45 (2.5)
Type of care for prescribed index drug, *N* (%)			
Primary	397 (34.0)	476 (75.7)	873 (48.6)
Secondary	23 (66.1)	22 (24.3)	45 (51.4)

*BMI* body mass index, *GP* general practitioner, *SD* standard deviation

^a^At index date

^b^Data based on 1185 patients (mirabegron: *N* = 790; antimuscarinics: *N* = 395)

^c^Data based on follow-up post-index date

### Persistence

#### All eligible patients

**Fig. 2:**
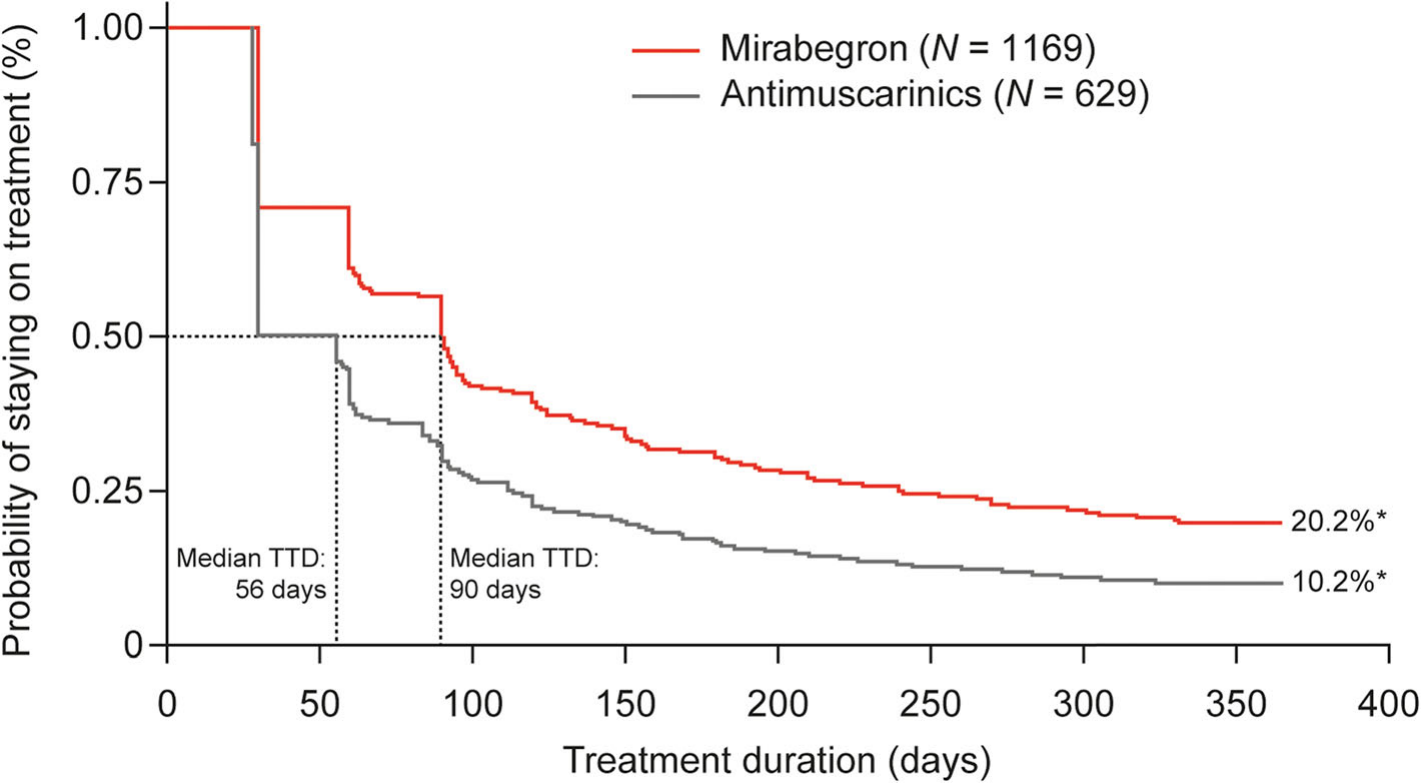
Median time to discontinuation for mirabegron versus antimuscarinics (base-case analysis; all eligible patients [*N* = 1798]). ^*^Proportion of patients persistent at 12 months. CI, confidence intervals; HR, hazard ratio; TTD, time to discontinuation

Median TTD was longer with mirabegron versus antimuscarinic therapy (90 vs 56 days, respectively (Fig. 2). A higher proportion of patients prescribed mirabegron were persistent at 12 months (*N* = 236; 20.2%) compared with antimuscarinics (*N* = 64; 10.2%).

#### Multivariate analyses

Patients who received mirabegron were significantly less likely to discontinue treatment compared with those prescribed an antimuscarinic (HR: 1.52 [95% CI: 1.37–1.70]; *p* < 0.001) (Table 2). Patients < 75 years of age and treatment-naïve patients were significantly more likely to discontinue treatment compared with those ≥75 years of age and treatment-experienced patients, respectively (Table 2).

**Table 2:** Impact of covariates on time to discontinuation: multivariate Cox regression analysis adjusting for baseline characteristics in all eligible patients (*N* = 1798)^a^

	Target OAB drug received		Treatment status		Age, years		
	Mirabegron (*N* = 1169)^b^	Antimuscarinics (*N* = 629)	Naïve (*N* = 1289)	Experienced (*N* = 509)^b^	45–64 (*N* = 649)	65–74 (*N* = 595)	≥75 (*N* = 554)^b^
TTD							
Median, days	90	56	60	99	60	90	91
(95% CI)	90–92	30–56	60–61	92–121	56–60	68–91	90–99
HR (95% CI)	–	1.52 (1.37–1.70)^c^	1.35 (1.20–1.53)^c^	–	1.53 (1.35–1.74)^c^	1.15 (1.01*–*1.31)^d^	–

*ATC* Anatomical Therapeutic Chemical, *CI* confidence intervals, *GP* general practitioner, *HR* hazard ratio, *OAB* overactive bladder, *TTD* time to discontinuation

^a^At index date; HR, 95% CI and *p*-values generated using a stepwise Cox regression model including target OAB drug received, treatment status and age

^b^Reference comparator

^c^*p* < 0.001

^d^*p* = 0.039 (all other comparisons non-significant)

#### Subgroup analyses

**Fig. 3:**
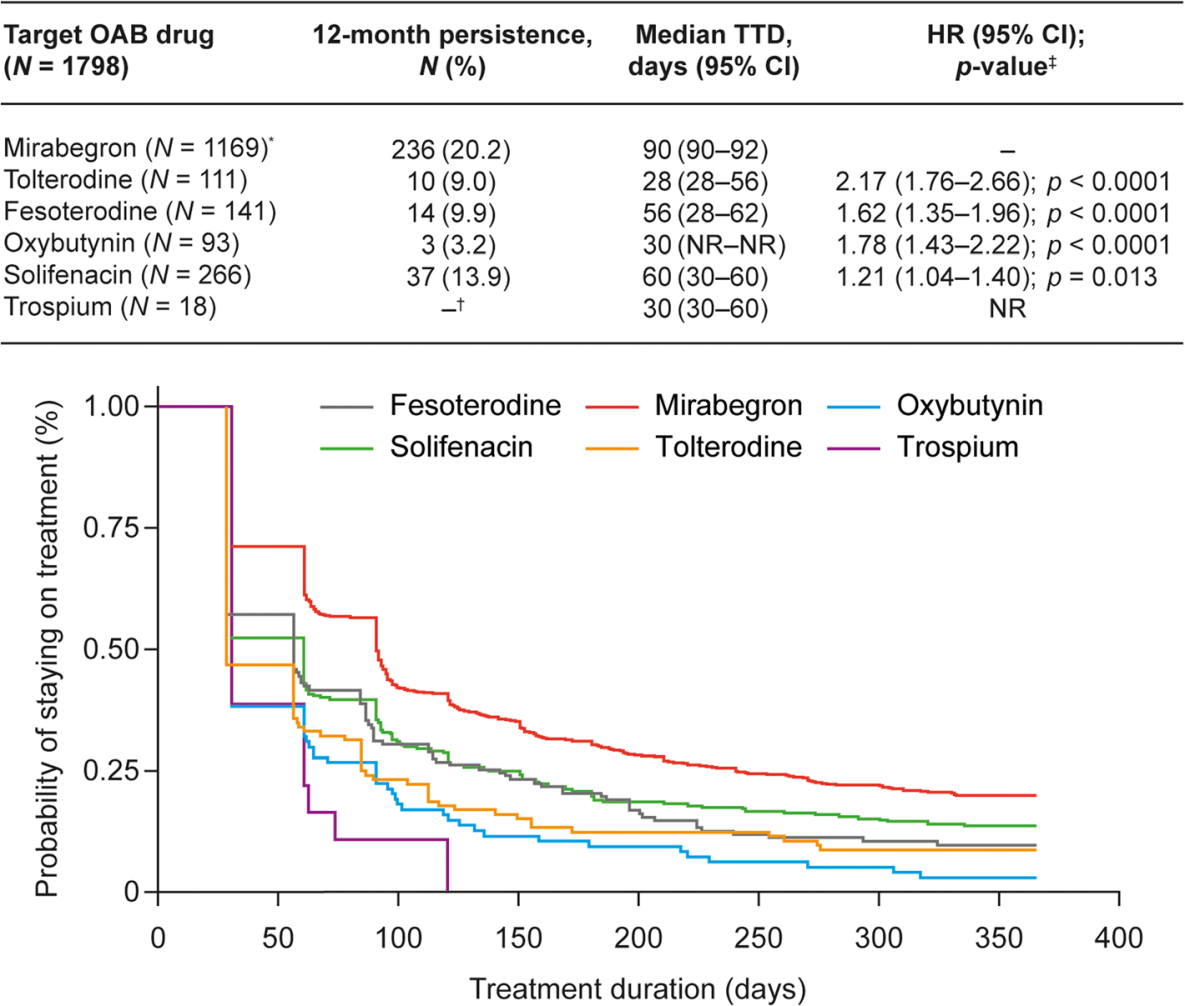
Persistence with mirabegron compared with individual antimuscarinics: subgroup analysis. ^*^Reference comparator; ^†^Not evaluated due to small sample size; ^‡^HRs, 95% CI and *p*-values generated using a Cox regression model with adjustment for age and treatment status. CI, confidence intervals; HR, hazard ratio; NR, not reached; OAB, overactive bladder; TTD, time to discontinuation

Median TTD for mirabegron (90 days) was significantly longer versus each individual antimuscarinic (range: 28–60 days; HR range: 1.21–2.17; *p* ≤ 0.013), and a higher proportion of patients were persistent at 12 months with mirabegron (20.2%) versus individual antimuscarinics (range: 3.2–13.9%) (Fig. 3). Among the antimuscarinics, solifenacin had the longest median TTD and the highest proportion of persistent patients. Treatment persistence was significantly longer with mirabegron versus all antimuscarinics in all the other subgroups: treatment naïve (HR: 1.47 [95% CI: 1.30–1.66]); treatment experienced (HR: 1.80 [95% CI: 1.41–2.31]); < 65 years (HR: 1.39 [95% CI: 1.17–1.69]); ≥65 years (HR: 1.60 [95% CI: 1.39–1.83]); male (HR: 1.75 [95% CI: 1.48–2.07]); and female (HR: 1.37 [95% CI: 1.18–1.58]) (*p* < 0.001 for all comparisons) (Additional file 3: Figure S2).

#### Sensitivity analyses

Median TTD in patients prescribed mirabegron was increased from 90 days in the base-case analysis to 121 days, when the period without prescription renewal used to define treatment discontinuation was extended from 30 to 90 days (Additional file 4: Figure S3). This analysis had little impact on median TTD in patients who received an antimuscarinic (range: 56–60 days across all adjusted periods); median TTD was significantly longer for mirabegron versus antimuscarinics across all of the adjusted time periods used to define treatment discontinuation (HR range: 1.43–1.53; *p* < 0.001 for all assessments).

### Adherence

#### All eligible patients

Mean adherence using fixed-MPR was higher with mirabegron (38.69 [SD: 33.75] versus antimuscarinics (25.88 [SD: 28.14]) and the proportion of adherent patients was two-times higher with mirabegron versus antimuscarinics (22.0% versus 11.0%, respectively). Adherence measured using variable-MPR was similar between the two groups (Table 3). The results of the multivariate and subgroup analyses of MPR are described in Additional file 5, with results displayed in Additional file 6: Tables S2, Additional file 7: Tables S3, Additional files 8: Table S4.

**Table 3:** Summary of adherence to mirabegron compared with antimuscarinics (all eligible patients; *N* = 1798)

	Mirabegron (*N* = 1169)	Antimuscarinics (*N* = 629)
MPR-fixed		
Mean (SD)	38.69 (33.75)	25.88 (28.14)
Adherent^a^, *N* (%)	257 (22.0)	69 (11.0)
MPR-variable		
Mean (SD)	97.65 (4.03)	98.03 (4.57)
Adherent^a^, *N* (%)	1163 (99.5)	619 (98.4)

*MPR* medical possession ratio, *SD* standard deviation

^a^MPR of ≥80%

## Discussion

This retrospective study assessed treatment persistence and adherence in approximately 1800 patients with OAB in Spain, who received mirabegron or an antimuscarinic in routine clinical practice. Greater persistence was observed for mirabegron versus antimuscarinics in both the unadjusted (all eligible patients) and adjusted (multivariate) analyses, including a two-times larger proportion of persistent and adherent patients at 12 months in the mirabegron arm (primary and secondary endpoints). These findings were supported by the results of the sensitivity analyses and the data reported in various patient subgroups.

Overall, the results of our study add to the growing body of real-world evidence from several other countries, which indicates mirabegron provides greater treatment persistence compared with antimuscarinics in patients with OAB [21–23, 32]; 12-month persistence with mirabegron ranged from 33% to 39% in three of these studies [21, 22, 32]. Recent evidence from BELIEVE, a large, prospective, non-interventional study of 682 patients from eight European countries (NCT02320773), reported that 53.8% of patients were persistent after 12 months on-treatment with mirabegron [39]; this was the highest 12-month persistence rate observed for mirabegron in real-world studies to date.

Median TTD and 12-month persistence rates for all OAB treatment reported in the current study were lower compared with recent retrospective, observational studies of treatment persistence with mirabegron and antimuscarinics conducted in different countries [21, 22, 40]. However, rates of 12-month persistence observed in the current study are comparable with some of the data reported in one other retrospective analysis (18%) [11, 23], and two systematic literature reviews of persistence in patients with OAB (12–39% and 10–25%) [12, 13]. National issues relating to clinical practice in Spain may have contributed to lower treatment persistence compared with some studies. For example, patients in Spain are expected to co-pay up-to 60% of drug costs [41]; cost is specified as one of several factors that might contribute to treatment discontinuation in the current European Association of Urology (EAU) guidelines for UI [9].

Two similar studies to evaluate persistence on-treatment in adults with OAB have been conducted in Spain, which reported higher rates of 12-month persistence on-treatment with solifenacin, fesoterodine or tolterodine monotherapy (range: 30.9–40.2%) compared with our study [34, 35]. These differences may be attributed to several factors: first, in both other studies, retrospective data were extracted from existing medical records from primary healthcare centres in parts of Catalonia and the Balearic Islands, compared with data representative of the overall Spanish population in our study. Second, our sensitivity analyses suggest that extending the period used to define treatment discontinuation results in greater persistence, and one of these other studies (Sicras-Mainar et al., 2014) used a considerably longer period to define treatment discontinuation (52 weeks without prescription renewal) versus our study (30 days in the base case analysis) [34]. In the other prior Spanish study (Sicras-Mainar et al., 2015), OAB patients between 20 and 64 years of age who were active workers were included; therefore, it’s possible these patients may have been healthier or had a better treatment response, compared with the patient population in our study which were selected using less-stringent criteria [35]. However, no stratification was performed to evaluate the potential impact of these patient characteristics.

A high proportion of patients included in our study (82.3%) were also overweight or obese according to BMI; these patients may be less likely to experience symptom improvement (and perhaps more likely to discontinue treatment) than those with lower BMI. Weight loss has been shown to reduce the number of UI episodes in overweight or obese women [42, 43], and is recommended by the EAU to reduce the risk of developing UI, or improve symptoms [9]. Similar to the current study, the mean BMI values in European patients with OAB enrolled in phase II/III studies were also indicative of overweight [19, 44, 45]; the impact of patients’ BMI on persistence and adherence was not evaluated in our study and this could perhaps be investigated in subsequent analyses.

In our study, the majority of patients (71.7%) were defined as treatment-naïve (i.e. they had not received any prescriptions for an OAB drug during the 12 months prior to the index date), which may have led to different expectations regarding efficacy and/or tolerability compared with treatment-experienced patients, and perhaps contributed to the observed low overall persistence. A proportionally smaller number of treatment-naïve patients were prescribed mirabegron vs antimuscarinics, and as reported in our study and other studies [21, 22, 46, 47], this may have had a positive impact on the persistence data – although treatment persistence was significantly longer with mirabegron versus all antimuscarinics in both the treatment naïve and treatment experienced subgroups. Most patients were prescribed antimuscarinics through primary care by a GP, while the majority of patients prescribed mirabegron received their prescription from a gynaecologist or urologist in secondary care. In the multivariate analysis of our study, the type of prescriber was not identified (during the stepwise selection process) as a covariate which produced a significant impact on TTD. However, we acknowledge there are two reports that indicate patients prescribed OAB treatment within a specialist setting experienced greater persistence compared with internal medicine/GP departments [48, 49].

Patients’ medication-taking behaviour can be attributed to many different factors and one of these is the nature of the communication with their physician [50]; others include the patient’s beliefs and values [50], expectations of treatment [14], perception of the severity of their condition [51], and other behavioural or societal factors [51]. To mitigate some of these factors having a negative impact on-treatment persistence and adherence, it is important that the patients’ treating physician provides them with the appropriate information/education during initial consultations, involves the patient in decision making throughout the course of treatment, and helps to manage their expectations around efficacy and tolerability. Also, a wide variety of comorbidities (including other urological conditions, neurological diseases, endocrine disorders, respiratory dysfunctions, faecal motility disorders, and pelvic cancer) may cause or worsen OAB symptoms [27]. Therefore, individualised treatment for patients with OAB, taking all aspects of a patients’ condition into account (i.e. treating OAB in the context of all comorbidities, rather than as a single condition) may be crucial for improving rates of persistence and adherence.

A higher proportion of patients in the current study (65.0%) received mirabegron compared with antimuscarinics (35.0%). Of the 629 patients prescribed an antimuscarinic, only trospium was prescribed to fewer than 90 patients and this antimuscarinic was not evaluated for 12-month persistence due to the small sample size (*N* = 18). The difference in the number of patients who received mirabegron and antimuscarinics may be attributed to mirabegron first being marketed in Spain in April 2014, fewer than two months prior to the start of the selection period [16]. It is possible that the proportional difference in prescriptions observed for mirabegron versus antimuscarinics may have contributed to the findings of our study, but this was not formally assessed.

Strengths of the study were the large sample size of approximately 1800 patients with OAB, and the collection of real-world data from a representative sample of patients treated in routine clinical practice in Spain. The main study limitation was the lack of significance estimates for the primary and secondary endpoints, which were not available as the protocol did not allow for adjustment of potential confounding factors in these specific analyses. However, significantly greater persistence was observed for mirabegron compared with antimuscarinics when Cox regression analyses were performed. Although the study tested for the confounding factors expected to be most important based on previous studies (and only those which had a significant association with TTD were included in our Cox regression models), the nature of retrospective database studies meant that all important confounding factors were unlikely to be captured, due to the analyses of limited secondary data (for example, information on the type of OAB symptoms or their severity were unavailable). Other limitations related to the database included persistence and adherence measured based on prescription data, rather than information being directly recorded by the treating physician; plus, several factors that could potentially impact treatment persistence were not recorded (e.g. physician compliance to management guidelines; severity of OAB symptoms; overall health/fragility of patients; physician bias for initial treatment/switching). Also, the trends observed for fixed- and variable-MPR were not consistent and might have resulted from a proxy of the methodology used (i.e. denominators of 365 days and median TTD, respectively). Finally, persistence and adherence estimates may not have been completely accurate, as the study evaluated prescriptions issued, but information regarding collection or correct use of the prescribed medication according to the recommended regimen was not available; however, this would have likely impacted all patients, independent of the OAB treatment prescribed.

It is possible that the observed differences in persistence and adherence for mirabegron versus antimuscarinics in the current study, consistent with previous studies conducted in Canada and the UK [21, 22], could be attributed to an improved tolerability profile with mirabegron; in particular, a lower incidence of bothersome anticholinergic AEs such as dry mouth and constipation [20, 52]. However, as discussed above, many different factors are involved in patients’ medication-taking behaviour. Recently published data from the BELIEVE study reported insufficient relief of symptoms, poor tolerability due to side effects, and the cost/amount of co-pay among the most common reasons for switching from or discontinuing treatment with mirabegron [39]. Future studies, including PERSPECTIVE (Clinicaltrials.gov: NCT02386072 [53] should offer additional insights into the underlying differences in treatment persistence and adherence across therapies used for OAB, possible reasons for treatment discontinuation and the potential impact on clinical practice (e.g. for clinical outcomes, economic impact and resource use).

## Conclusions

Mirabegron is associated with greater treatment persistence and adherence in patients with OAB, compared with antimuscarinics. The differences in mechanism of action and efficacy/tolerability profile may contribute to this reported treatment benefit and supports data showing that mirabegron is an appropriate treatment option for patients with OAB. The real-world evidence from this longitudinal, observational cohort study of OAB in Spain may provide strategic insights for clinicians and policy makers involved in the management of patients with this chronic condition. However, further study is needed to understand the different factors that may contribute to low persistence and adherence on-treatment with OAB pharmacotherapy. Some of these factors may be mitigated by the patient’s treating physician providing them with appropriate information during initial consultations, involving them in decision making throughout the course of treatment, helping to manage their expectations around efficacy and tolerability, and implementing an individualised treatment strategy.

## Additional files

Additional file 1: **Figure S1.** Study design. (TIF 1446 kb)

Additional file 2: **Table S1.** Drugs available for selection using European Pharmaceutical Market Research Association (EphMRA) Anotomical Therapeutic Chemical (ATC) drug codes (1). (DOCX 14 kb)

Additional file 3: **Figure S2.** Median TTD for mirabegron versus antimuscarinics (base-case analysis) in the following subgroups of patients: treatment-naïve (A); treatment-experienced (B); < 65 years of age (C); ≥65 years of age (D); male (E); and female (F). (TIF 2887 kb)

Additional file 4: **Figure S3.** Median TTD when the 30-day period without prescription renewal used to define TTD was extended to 45 days (A); 60 days (B); and 90 days (C) (all eligible patients; N = 1798). (TIF 2722 kb)

Additional file 5 Summary of multivariate and subgroup analyses of MPR. (DOCX 16 kb)

Additional file 6: **Table S2.** Impact of covariates on the medical possession ratio: multivariate linear regression analysis in all eligible patients (*N* = 1798). (DOCX 17 kb)

Additional file 7: **Table S3.** Summary of adherence with mirabegron compared with antimuscarinics in subgroups defined by the target OAB drug received. (DOCX 17 kb)

Additional file 8: **Table S4.** Summary of adherence with mirabegron compared with antimuscarinics in subgroups defined by treatment experience, age and gender. (DOCX 17 kb)

## Abbreviations

AE: Adverse events
ATC: Anatomical Therapeutic Chemical
BMI: Body mass index
CI: Confidence intervals
EAU: European Association of Urology
EphMRA: European Pharmaceutical Market Research Association
GP: General practitioner
HR: Hazard ratio
HRQoL: Health-related quality of life
MPR: Medical possession ratio
NR: Not reached
OAB: Overactive bladder
SD: Standard deviation
SLR: Systematic literature review
TTD: Time to discontinuation
UI: Urinary incontinence
UK: United Kingdom

## References

[1] Abrams P, Cardozo L, Fall M, Griffiths D, Rosier P, Ulmsten U (2002). The standardisation of terminology in lower urinary tract function: report from the standardisation sub-committee of the international continence society. Urology.

[2] Haylen BT, de Ridder D, Freeman RM, Swift SE, Berghmans B, Lee J (2010). An international Urogynecological association (IUGA)/international continence society (ICS) joint report on the terminology for female pelvic floor dysfunction. Neurourol Urodyn.

[3] Castro D, Espuna M, Prieto M, Badia X (2005). Prevalence of overactive bladder in Spain: a population-based study. Arch Esp Urol.

[4] Stewart WF, Van Rooyen JB, Cundiff GW, Abrams P, Herzog AR, Corey R (2003). Prevalence and burden of overactive bladder in the United States. World J Urol.

[5] Nicolson P, Kopp Z, Chapple CR, Kelleher C (2008). It's just the worry about not being able to control it! A qualitative study of living with overactive bladder. Br J Health Psychol.

[6] Coyne KS, Sexton CC, Irwin DE, Kopp ZS, Kelleher CJ, Milsom I (2008). The impact of overactive bladder, incontinence and other lower urinary tract symptoms on quality of life, work productivity, sexuality and emotional well-being in men and women: results from the EPIC study. BJU Int.

[7] Irwin DE, Mungapen L, Milsom I, Kopp Z, Reeves P, Kelleher C (2009). The economic impact of overactive bladder syndrome in six western countries. BJU Int.

[8] Rapariz M, Mora AM, Roset M. Impact of overactive bladder symptoms on work activity: The ACTIVHA study. Actas Urol Esp. 2017; 10.1016/jacuro201709005. [Epub ahead of print] 2017 10.1016/j.acuro.2017.09.005 29103735

[9] Burkhard FC, Bosch JLHR, Cruz F, Lemack GE, Nambiar AK, Thiruchelvam N, Tubaro A. Guidelines on Urinary Incontinence. Available at: http://uroweb.org/guideline/urinary-incontinence/ (Access date: 27 Mar 2017).

[10] Cramer JA, Roy A, Burrell A, Fairchild CJ, Fuldeore MJ, Ollendorf DA (2008). Medication compliance and persistence: terminology and definitions. Value Health.

[11] Yeaw J, Benner JS, Walt JG, Sian S, Smith DB (2009). Comparing adherence and persistence across 6 chronic medication classes. J Manag Care Pharm.

[12] Veenboer PW, Bosch JL (2014). Long-term adherence to antimuscarinic therapy in everyday practice: a systematic review. J Urol.

[13] Sexton CC, Notte SM, Maroulis C, Dmochowski RR, Cardozo L, Subramanian D (2011). Persistence and adherence in the treatment of overactive bladder syndrome with anticholinergic therapy: a systematic review of the literature. Int J Clin Pract.

[14] Benner JS, Nichol MB, Rovner ES, Jumadilova Z, Alvir J, Hussein M (2010). Patient-reported reasons for discontinuing overactive bladder medication. BJU Int.

[15] European Medicines Agency. Betmiga EPAR Product Information. Available at: http://www.ema.europa.eu/ema/index.jsp?curl=pages/medicines/human/medicines/002388/human_med_001605.jsp&mid=WC0b01ac058001d124/ (Access date: 16 May 2017).

[16] BOTPLUS. National Spanish database from pharmacology colleges. Available at: https://botplusweb.portalfarma.com/botplus.aspx/ (Access date: 13 December 2017).

[17] Herschorn S, Barkin J, Castro-Diaz D, Frankel JM, Espuna-Pons M, Gousse AE (2013). A phase III, randomized, double-blind, parallel-group, placebo-controlled, multicentre study to assess the efficacy and safety of the beta(3) adrenoceptor agonist, mirabegron, in patients with symptoms of overactive bladder. Urology.

[18] Nitti VW, Auerbach S, Martin N, Calhoun A, Lee M, Herschorn S (2013). Results of a randomized phase III trial of mirabegron in patients with overactive bladder. J Urol.

[19] Khullar V, Amarenco G, Angulo JC, Cambronero J, Hoye K, Milsom I (2013). Efficacy and tolerability of mirabegron, a beta(3)-adrenoceptor agonist, in patients with overactive bladder: results from a randomised European-Australian phase 3 trial. Eur Urol.

[20] Maman K, Aballea S, Nazir J, Desroziers K, Neine ME, Siddiqui E (2014). Comparative efficacy and safety of medical treatments for the management of overactive bladder: a systematic literature review and mixed treatment comparison. Eur Urol.

[21] Wagg A, Franks B, Ramos B, Berner T (2015). Persistence and adherence with the new beta-3 receptor agonist, mirabegron, versus antimuscarinics in overactive bladder: early experience in Canada. Can Urol Assoc J.

[22] Chapple CR, Nazir J, Hakimi Z, Bowditch S, Fatoye F, Guelfucci F (2017). Persistence and adherence with mirabegron versus antimuscarinic agents in patients with overactive bladder: a retrospective observational study in UK clinical practice. Eur Urol.

[23] Nitti VW, Rovner ES, Franks B, Muma NM, Berner T, Fan A (2016). Persistence with mirabegron versus tolterodine in patients with overactive bladder. Am J Pharm Benefits.

[24] Athanasopoulos A, Giannitsas K (2011). An overview of the clinical use of antimuscarinics in the treatment of overactive bladder. Adv Urol.

[25] Chapple CR, Fianu-Jonsson A, Indig M, Khullar V, Rosa J, Scarpa RM (2007). Treatment outcomes in the STAR study: a subanalysis of solifenacin 5 mg and tolterodine ER 4 mg. Eur Urol.

[26] Chapple CR, Khullar V, Gabriel Z, Muston D, Bitoun CE, Weinstein D (2008). The effects of antimuscarinic treatments in overactive bladder: an update of a systematic review and meta-analysis. Eur Urol.

[27] Corcos J, Przydacz M, Campeau L, Gray G, Hickling D, Honeine C (2017). CUA guideline on adult overactive bladder. Can Urol Assoc J.

[28] Campbell UB, Stang P, Barron R (2008). Survey assessment of continuation of and satisfaction with pharmacological treatment for urinary incontinence. Value Health.

[29] Dmochowski RR, Newman DK (2007). Impact of overactive bladder on women in the United States: results of a national survey. Curr Med Res Opin.

[30] Andy UU, Arya LA, Smith AL, Propert KJ, Bogner HR, Colavita K (2015). Is self-reported adherence associated with clinical outcomes in women treated with anticholinergic medication for overactive bladder?. Neurourol Urodyn.

[31] Kim TH, Choo MS, Kim YJ, Koh H, Lee KS (2016). Drug persistence and compliance affect patient-reported outcomes in overactive bladder syndrome. Qual Life Res.

[32] Hakimi Z, Nazir J, McCrea C, Berling M, Fatoye F, Ramos B (2017). Clinical and economic impact of mirabegron compared with antimuscarinics for the treatment of overactive bladder in Canada. J Med Econ.

[33] Nazir J, Berling M, McCrea C, Fatoye F, Bowditch S, Hakimi Z (2017). Economic impact of mirabegron versus antimuscarinics for the treatment of overactive bladder in the UK. PharmacoEconomics - Open.

[34] Sicras-Mainar A, Rejas J, Navarro-Artieda R, Aguado-Jodar A, Ruiz-Torrejon A, Ibanez-Nolla J (2014). Antimuscarinic persistence patterns in newly treated patients with overactive bladder: a retrospective comparative analysis. Int Urogynecol J.

[35] Sicras-Mainar A, Navarro-Artieda R, Ruiz-Torrejon A, Saez-Zafra M, Coll-de Tuero G (2015). Impact of loss of work productivity in patients with overactive bladder treated with antimuscarinics in Spain: study in routine clinical practice conditions. Clin Drug Investig.

[36] World Health Organization. ATC/DDD Index 2017. Drugs for urinary frequency and incontinence. Available at: https://www.whocc.no/atc_ddd_index/?code=G04BD (Access date: 11 May 2017).

[37] Krall JM, Uthoff VA, Harley JB (1975). A step-up procedure for selecting variables associated with survival. Biometrics.

[38] Helmreich JE. Regression modeling strategies with applications to linear models, logistic and ordinal regression and survival analysis (2nd Edition). J Stat Softw. 2016;70(Book Review 2).

[39] Freeman R, Foley S, Rosa J, Vicente E, Grill R, Kachlirova Z, et al. Mirabegron improves quality of life, treatment satisfaction and persistence in patients with overactive bladder - a multicentre, non-interventional, real-world, 12-month study. Curr Med Res Opin. 2017:1–9. 10.1080/03007995. [Epub ahead of print] 10.1080/03007995.2017.1419170 29254376

[40] Wagg A, Compion G, Fahey A, Siddiqui E (2012). Persistence with prescribed antimuscarinic therapy for overactive bladder: a UK experience. BJU Int.

[41] Errando-Smet C, Muller-Arteaga C, Hernandez M, Lenero E, Roset M. Healthcare resource utilization and cost among males with lower urinary tract symptoms with a predominant storage component in Spain: the epidemiological, cross-sectional MERCURY study. Neurourol Urodyn. 2017; 10.1002/nau.23293. [epub ahead of print] 10.1002/nau.23293 28464366

[42] Subak LL, Johnson C, Whitcomb E, Boban D, Saxton J, Brown JS (2002). Does weight loss improve incontinence in moderately obese women?. Int Urogynecol J Pelvic Floor Dysfunct.

[43] Gozukara YM, Akalan G, Tok EC, Aytan H, Ertunc D (2014). The improvement in pelvic floor symptoms with weight loss in obese women does not correlate with the changes in pelvic anatomy. Int Urogynecol J.

[44] Malone-Lee JG, Walsh JB, Maugourd MF (2001). Tolterodine: a safe and effective treatment for older patients with overactive bladder. J Am Geriatr Soc.

[45] Abrams P, Kelleher C, Staskin D, Rechberger T, Kay R, Martina R (2015). Combination treatment with mirabegron and solifenacin in patients with overactive bladder: efficacy and safety results from a randomised, double-blind, dose-ranging, phase 2 study (symphony). Eur Urol.

[46] Kim TH, Lee KS (2016). Persistence and compliance with medication management in the treatment of overactive bladder. Investig Clin Urol.

[47] Wagg AS, Foley S, Peters J, Nazir J, Kool-Houweling L, Scrine L. Persistence and adherence with mirabegron vs antimuscarinics in overactive bladder: retrospective analysis of a UK general practice prescription database. Int J Clin Pract. 2017;71 10.1111/ijcp.12996 28906080

[48] Kalder M, Pantazis K, Dinas K, Albert US, Heilmaier C, Kostev K (2014). Discontinuation of treatment using anticholinergic medications in patients with urinary incontinence. Obstet Gynecol.

[49] Tran AM, Sand PK, Seitz MJ, Gafni-Kane A, Zhou Y, Botros SM (2017). Does physician specialty affect persistence to pharmacotherapy among patients with overactive bladder syndrome?. Int Urogynecol J.

[50] Shingler SL, Bennett BM, Cramer JA, Towse A, Twelves C, Lloyd AJ (2014). Treatment preference, adherence and outcomes in patients with cancer: literature review and development of a theoretical model. Curr Med Res Opin.

[51] Touchette D, Shapiro N (2008). Medication compliance, adherence, and persistence: current status of behavioral and educational interventions to improve outcomes. J Manag Care Pharm.

[52] Nitti VW, Khullar V, van Kerrebroeck P, Herschorn S, Cambronero J, Angulo JC (2013). Mirabegron for the treatment of overactive bladder: a prespecified pooled efficacy analysis and pooled safety analysis of three randomised, double-blind, placebo-controlled, phase III studies. Int J Clin Pract.

[53] Clinicaltrials.gov. A Prospective, Observational, Multicenter Study of Patients Following Initiation of a New Course of Treatment for Overactive Bladder (OAB) (PERSPECTIVE). Available at: https://clinicaltrials.gov/ct2/show/NCT02386072?term=persistence+AND+overactive+bladder&rank=7/ (Access date: 19 May 2017).

