# Supplementary figures and images for: A retrospective study of treatment persistence and adherence to mirabegron versus antimuscarinics, for the treatment of overactive bladder in Spain
Source: BMC Urol. 2018 Sep 4;18:76. doi: 10.1186/s12894-018-0390-z (PMC6122705; doi:10.1186/s12894-018-0390-z)

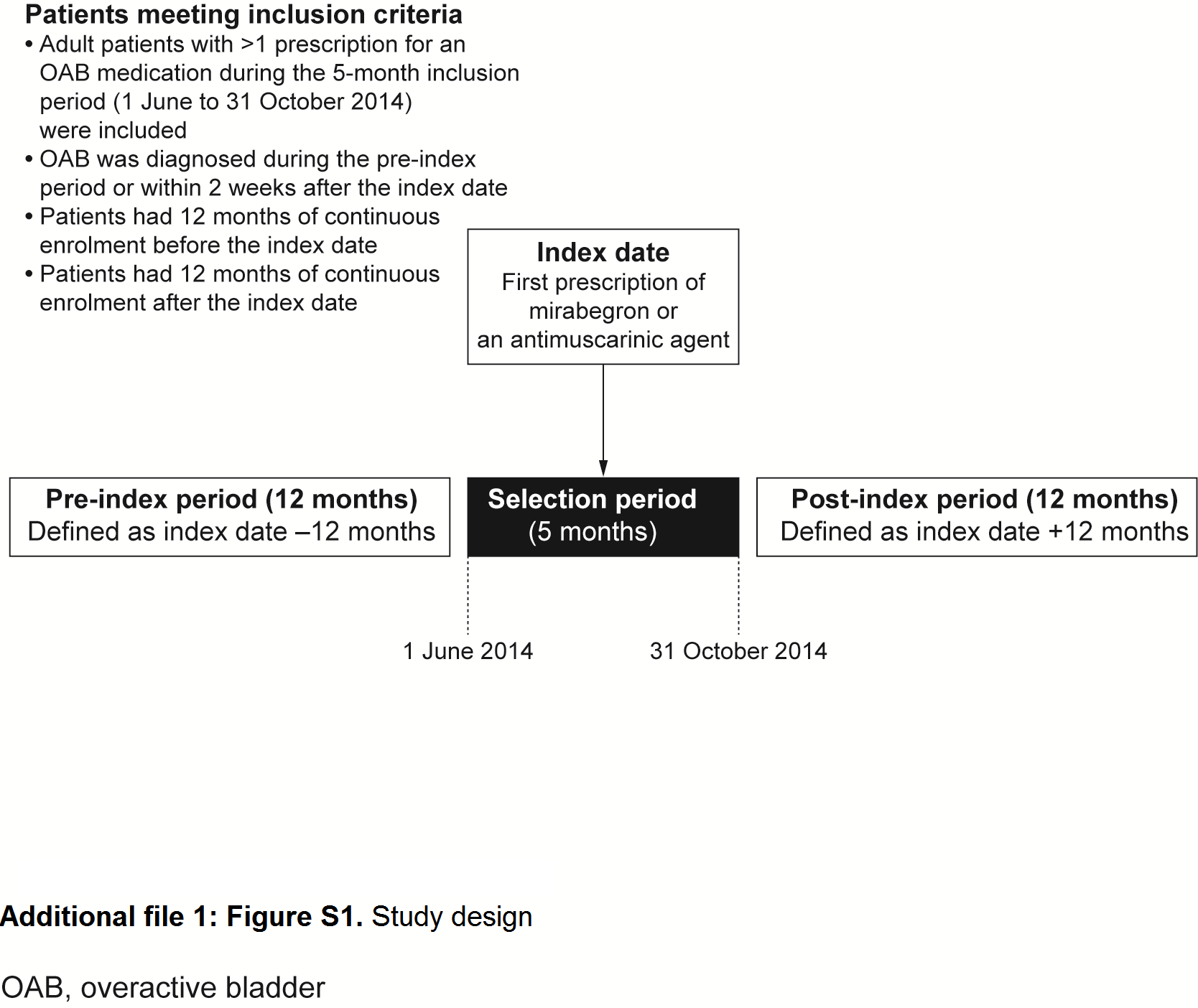

Supplement: Supplementary file 1 — Figure S1. Study design. (TIF 1446 kb) [file 12894_2018_390_MOESM1_ESM.tif]

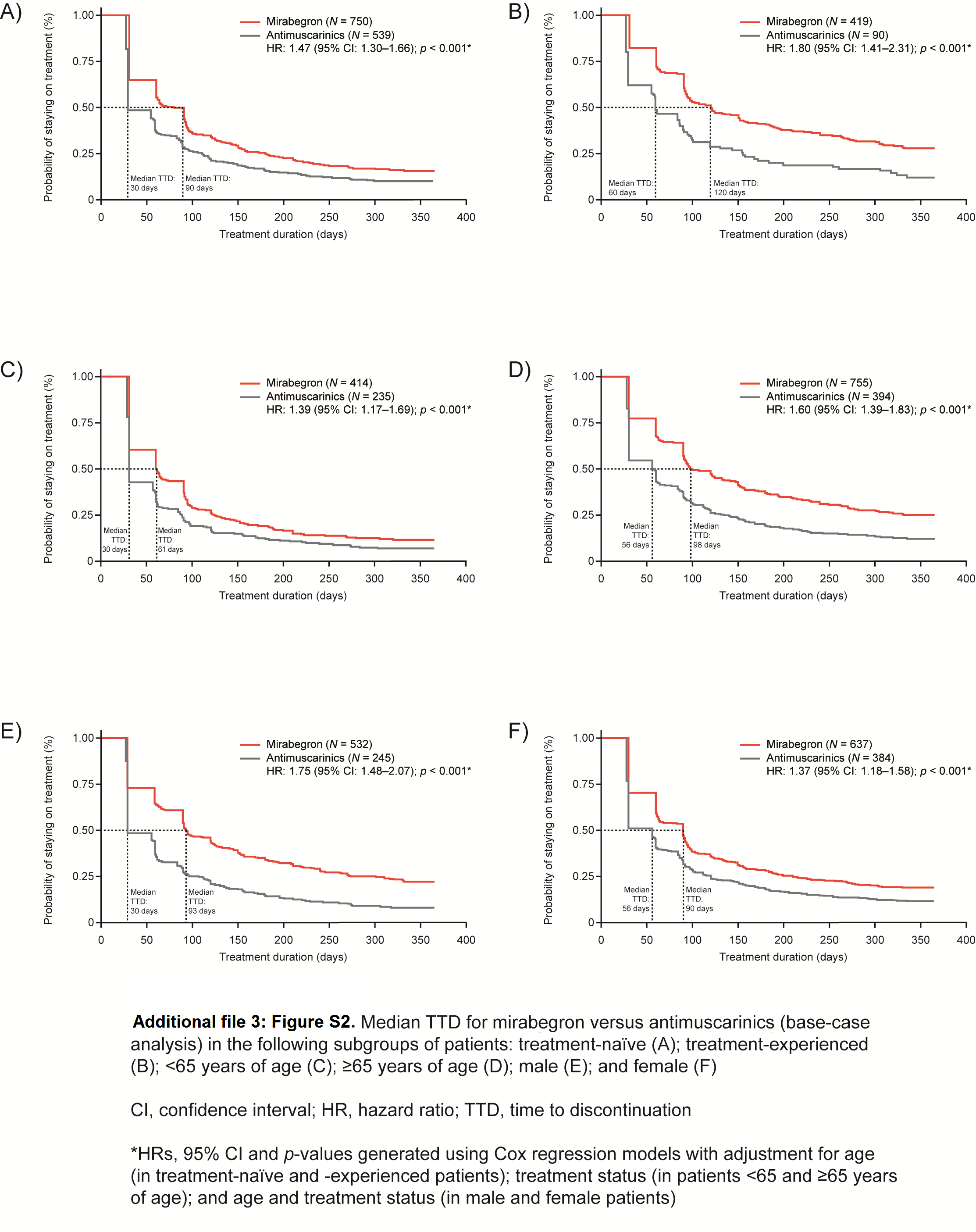

Supplement: Supplementary file 3 — Figure S2. Median TTD for mirabegron versus antimuscarinics (base-case analysis) in the following subgroups of patients: treatment-naïve (A); treatment-experienced (B); < 65 years of age (C); ≥65 years of age (D); male (E); and female (F). (TIF 2887 kb) [file 12894_2018_390_MOESM3_ESM.tif]

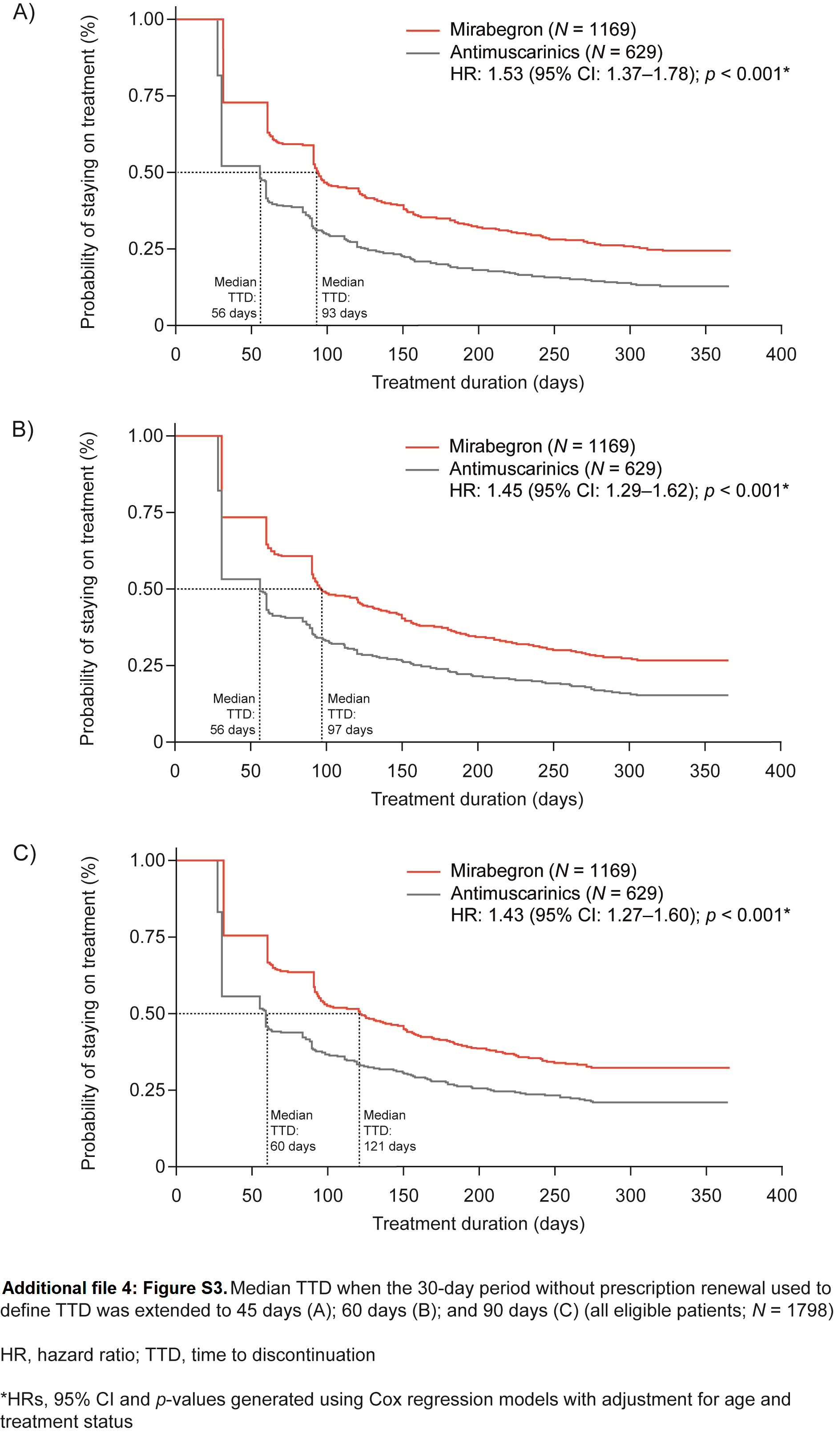

Supplement: Supplementary file 4 — Figure S3. Median TTD when the 30-day period without prescription renewal used to define TTD was extended to 45 days (A); 60 days (B); and 90 days (C) (all eligible patients; N = 1798). (TIF 2722 kb) [file 12894_2018_390_MOESM4_ESM.tif]
