# Supplementary material for: A retrospective study of treatment persistence and adherence to mirabegron versus antimuscarinics, for the treatment of overactive bladder in Spain
Source: BMC Urol. 2018 Sep 4;18:76. doi: 10.1186/s12894-018-0390-z (PMC6122705; doi:10.1186/s12894-018-0390-z)
Supplement: Supplementary file 2 — Table S1. Drugs available for selection using European Pharmaceutical Market Research Association (EphMRA) Anotomical Therapeutic Chemical (ATC) drug codes (1). (DOCX 14 kb) [file 12894_2018_390_MOESM2_ESM.docx]

**Additional file 2: Table S1.** Drugs available for selection using European Pharmaceutical Market Research Association (EphMRA) Anotomical Therapeutic Chemical (ATC) drug codes (1)^a^

| Drug | ATC code |
| --- | --- |
| Antimuscarinic |  |
| Emepronium | G04BD01 |
| Flavoxate | G04BD02 |
| Meladrazine | G04BD03 |
| Oxybutynin | G04BD04 |
| Terodiline | G04BD05 |
| Propiverine | G04BD06 |
| Tolterodine | G04BD07 |
| Solifenacin | G04BD08 |
| Trospium | G04BD09 |
| Darifenacin | G04BD10 |
| Fesoterodine | G04BD11 |
| β_3‑_adrenoceptor agonist |  |
| Mirabegron | G04BD12 |

*ATC* Anatomical Therapeutic Chemical, *EphMRA* European Pharmaceutical Market Research Association

^a^Drugs listed were included in all database searches, but some are not marketed in Spain and were therefore not included in the analyses of persistence and adherence

Reference

1. World Health Organization. ATC/DDD Index 2017. Drugs for urinary frequency and incontinence. Available at: <https://www.whocc.no/atc_ddd_index/?code=G04BD/> (access date: 11 May 2017).
