# Supplementary material for: A retrospective study of treatment persistence and adherence to mirabegron versus antimuscarinics, for the treatment of overactive bladder in Spain
Source: BMC Urol. 2018 Sep 4;18:76. doi: 10.1186/s12894-018-0390-z (PMC6122705; doi:10.1186/s12894-018-0390-z)
Supplement: Supplementary file 5 — Summary of multivariate and subgroup analyses of MPR. (DOCX 16 kb) [file 12894_2018_390_MOESM5_ESM.docx]

**Additional file 5:** Summary of multivariate and subgroup analyses of MPR

*Multivariate analyses*

Adherence assessed by fixed-MPR was significantly greater for mirabegron versus antimuscarinics
(*p* < 0.001); treatment-experienced versus -naïve (*p* < 0.001); and ≥65 versus <65 years of age
(*p* < 0.001) (**Table S2**). No notable differences were observed for mirabegron versus antimuscarinics or treatment-experienced versus -naïve patients, when adherence was assessed using variable-MPR (**Table S2**).

*Subgroup analyses*

Using fixed-MPR, a significantly higher mean adherence and proportion of adherent patients were observed for mirabegron versus each individual antimuscarinic (**Table S3**). Significantly higher mean adherence was reported for mirabegron versus antimuscarinics in each of the other six subgroups assessed, and the proportion of adherent patients was significantly higher for mirabegron versus antimuscarinics in 4/6 of these subgroups (treatment-naïve and -experienced, male, and
<65-year old patients; **Table S4**).
