# Supplementary material for: A retrospective study of treatment persistence and adherence to mirabegron versus antimuscarinics, for the treatment of overactive bladder in Spain
Source: BMC Urol. 2018 Sep 4;18:76. doi: 10.1186/s12894-018-0390-z (PMC6122705; doi:10.1186/s12894-018-0390-z)
Supplement: Supplementary file 6 — Table S2. Impact of covariates on the medical possession ratio: multivariate linear regression analysis in all eligible patients (N = 1798). (DOCX 17 kb) [file 12894_2018_390_MOESM6_ESM.docx]

# **Additional file 6: Table S2.** Impact of covariates on the medical possession ratio: multivariate linear regression analysis in all eligible patients (*N* = 1798)

|  | Target OAB drug received | | Treatment status | | Age, years | |
| --- | --- | --- | --- | --- | --- | --- |
|  | **Mirabegron^a^**  **(*N* = 1169)** | **Antimuscarinics**  **(*N* = 629)** | **Treatment-naïve**  **(*N* = 1289)** | **Treatment-experienced^a^**  **(*N* = 509)** | **<65^a^**  **(*N* = 649)** | **≥65**  **(*N* = 1149)** |
| MPR-fixed |  |  |  |  |  |  |
| Estimate (SE) | – | –10.64 (1.73) | –10.91 (1.68) |  | – | 11.36 (1.55) |
| *p*-value^b^ |  | < 0.001 | < 0.001 |  |  | < 0.001 |
| MPR-variable |  |  |  |  |  |  |
| Estimate (SE) | – | 0.26 (0.21) | 0.55 (0.23) |  | – | |
| *p*-value^b^ |  | 0.221 | 0.016 |  |  |  |

*MPR* medical possession ratio, *SE* standard error

^a^Reference comparator

^b^*p-*values generated using a linear regression model with adjustment for treatment status and age (fixed-MPR); and adjustment for treatment status (variable MPR)
