# Supplementary material for: A retrospective study of treatment persistence and adherence to mirabegron versus antimuscarinics, for the treatment of overactive bladder in Spain
Source: BMC Urol. 2018 Sep 4;18:76. doi: 10.1186/s12894-018-0390-z (PMC6122705; doi:10.1186/s12894-018-0390-z)
Supplement: Supplementary file 7 — Table S3. Summary of adherence with mirabegron compared with antimuscarinics in subgroups defined by the target OAB drug received. (DOCX 17 kb) [file 12894_2018_390_MOESM7_ESM.docx]

**Additional file 7: Table S3.** Summary of adherence with mirabegron compared with antimuscarinics in subgroups defined by the target OAB drug received

|  | Mirabegron  (*N* = 1169) | Tolterodine  (*N* = 111) | Fesoterodine  (*N* = 141) | Oxybutynin  (*N* = 93) | Solifenacin  (*N* = 266) | Trospium  (*N* = 18) | Total  (*N* = 1798) |
| --- | --- | --- | --- | --- | --- | --- | --- |
| MPR-fixed |  |  |  |  |  |  |  |
| Mean (SD) | 38.69 (33.75) | 23.08 (27.40)^c^ | 27.48 (28.03)^c^ | 18.92 (20.06)^c^ | 29.49 (30.98)^c^ | 13.21 (8.02)^c^ | 34.21 (32.47) |
| Adherent^a^, *N* (%) | 257 (22.0) | 10 (9.0)^c^ | 15 (10.6)^b^ | 3 (3.2)^c^ | 41 (15.4)^b^ | 0^b^ | 326 (18.1) |
| MPR-variable |  |  |  |  |  |  |  |
| Mean (SD) | 97.65 (4.03) | 97.71 (6.54) | 98.07 (3.63) | 97.52 (5.35) | 98.26 (3.68)^b^ | 98.83 (4.22) | 97.78 (4.23) |
| Adherent^a^, *N* (%) | 1163 (99.5) | 107 (96.4)^b^ | 140 (99.3) | 90 (96.8)^b^ | 264 (99.2) | 18 (100) | 1782 (99.1) |

*MPR* medical possession ratio; *SD* standard deviation

^a^MPR of ≥80%;

Difference between mirabegron and antimuscarinics: ^b^*p* < 0.05; ^c^*p* < 0.001 (all other comparisons non-significant); *p*-values generated using a linear regression model with adjustment for gender, age, treatment status and clinical speciality (fixed-MPR); and adjustment for treatment status (variable-MPR)
