# Supplementary material for: A retrospective study of treatment persistence and adherence to mirabegron versus antimuscarinics, for the treatment of overactive bladder in Spain
Source: BMC Urol. 2018 Sep 4;18:76. doi: 10.1186/s12894-018-0390-z (PMC6122705; doi:10.1186/s12894-018-0390-z)
Supplement: Supplementary file 8 — Table S4. Summary of adherence with mirabegron compared with antimuscarinics in subgroups defined by treatment experience, age and gender. (DOCX 17 kb) [file 12894_2018_390_MOESM8_ESM.docx]

**Additional file 8: Table S4.** Summary of adherence with mirabegron compared with antimuscarinics in subgroups defined by treatment experience, age and gender

|  | Treatment-naïve  patients | | Treatment-experienced patients | | Patients <65 years | | Patients ≥65 years | | Male patients | | Female patients | |
| --- | --- | --- | --- | --- | --- | --- | --- | --- | --- | --- | --- | --- |
|  | **Mirabegron**  **(*N* = 750)** | **AMs**  **(*N* = 539)** | **Mirabegron**  **(*N* = 419)** | **AMs**  **(*N* = 90)** | **Mirabegron**  **(*N* = 414)** | **AMs**  **(*N* = 235)** | **Mirabegron**  **(*N* = 755)** | **AMs**  **(*N* = 394)** | **Mirabegron**  **(*N* = 532)** | **AMs**  **(*N* = 245)** | **Mirabegron**  **(*N* = 637)** | **AMs**  **(*N* = 384)** |
| MPR-fixed |  |  |  |  |  |  |  |  |  |  |  |  |
| Mean (SD) | 33.82 (31.76) | 25.01 (27.58)^c^ | 47.41 (35.46) | 31.08 (30.91)^c^ | 28.72 (28.61) | 21.48 (24.54)^b^ | 44.16 (35.10) | 28.50 (29.80)^c^ | 41.35 (34.59) | 23.70 (25.74)^c^ | 36.47 (32.90) | 27.27 (29.51)^c^ |
| Adherent^a^, *N* (%) | 128 (17.1) | 55 (10.2)^c^ | 129 (30.8) | 14 (15.6)^b^ | 51 (12.3) | 17 (7.2) | 206 (27.3) | 52 (13.2)^c^ | 132 (24.8) | 20 (8.2)^c^ | 125 (19.6) | 49 (12.8) |
| MPR-variable |  |  |  |  |  |  |  |  |  |  |  |  |
| Mean (SD) | 97.86 (3.89) | 98.10 (4.57) | 97.28 (4.25) | 97.64 (4.58) | 97.72 (4.23) | 98.27 (4.17) | 97.61 (3.92) | 97.89 (4.80) | 97.77 (3.93) | 98.09 (4.54) | 97.55 (4.11) | 97.99 (4.60) |
| Adherent^a^, *N* (%) | 749 (99.9) | 530 (98.3)^b^ | 414 (98.8) | 89 (98.9) | 411 (99.3) | 232 (98.7) | 752 (99.6) | 387 (98.2)^b^ | 531 (99.8) | 241 (98.4) | 632 (99.2) | 378 (98.4) |

*AMs* antimuscarinics, *MPR* medical possession ratio; *SD* standard deviation

^a^MPR of ≥80%

Difference between mirabegron and antimuscarinics: ^b^*p* ≤ 0.05; ^c^*p* < 0.001 (all other comparisons non-significant); *p*-values generated using a linear regression model with adjustment for age and treatment status (fixed-MPR); and adjustment for treatment status (variable-MPR)
